# Quality of life in patients with Fabry disease: a systematic review of the literature

**DOI:** 10.1186/s13023-015-0296-8

**Published:** 2015-06-16

**Authors:** Maarten Arends, Carla E. M. Hollak, Marieke Biegstraaten

**Affiliations:** Department of Internal Medicine, Division Endocrinology and Metabolism, Academic Medical Center, PO Box 22660, Amsterdam, 1100 DD The Netherlands

**Keywords:** Fabry disease, Quality of life, Health related quality of life, Patient reported outcome measures, Enzyme replacement therapy, Systematic review

## Abstract

Fabry disease (FD), caused by deficiency of the lysosomal enzyme α-galactosidase-A, is a progressive multisystem disease. The disease is X-linked with generally more severe manifestations in males, but can impact on quality of life (QoL) of both male and female patients. The purpose of this literature review is to analyse the currently available data concerning QoL measurement, specifically which questionnaires have been used to measure QoL, how patients with FD score compared to the general population, and the effects of enzyme replacement therapy (ERT) on QoL. Fifty-four articles were relevant for this literature review. Patients with FD had a lower QoL compared to the general population. No definite conclusions could be drawn from the studies on the effect of ERT on QoL; natural history data is scarce, changes observed were limited and the cohorts were of small size. We propose that a FD specific questionnaire be made to accurately assess QoL in patients with FD.

**Electronic supplementary material:**

The online version of this article (doi:10.1186/s13023-015-0296-8) contains supplementary material, which is available to authorized users.

## Introduction

Fabry disease (FD) (OMIM#301500) is a rare X-linked lysosomal storage disorder. The disease is characterized by deficiency of the lysosomal enzyme α-galactosidase A (α-Gal A, E.C. 3.2.1.22). This results in a systemic accumulation of globotriaosylceramide (Gb3) and related glycosphingolipids in lysosomes in cells throughout the body. The prevalence of FD is estimated at 1:40.000–170.000 live births [1–3] although recent newborn and high-risk group screening studies suggested that the prevalence of non-classical FD may be much higher than previously thought [4, 5]. Phenotypically, FD can be distinguished in the more severe classical form of FD, predominantly affecting males, and a non-classical form, more prominent in males with residual enzyme activity. Although females can be as severely affected as male patients with classical FD, most of them have a more variable and attenuated phenotype and are therefore better characterised as non-classical patients [6].

Early symptoms in classically affected male and female patients include angiokeratoma, anhydrosis, neuropathic pain, gastrointestinal symptoms and microalbuminuria. Later in life, progressive renal failure, heart failure and stroke generally occur. In non-classically affected male patients and most females, the disease presents with a more attenuated and variable disease course [7–11]. The shortened life expectancy and the morbidity of Fabry patients are strongly related to the degree of end-organ damage.

Currently, two enzyme preparations are available for the treatment of FD (agalsidase alfa, Shire HGT, Boston MA, USA, and agalsidase beta, Genzyme Inc, Boston MA, USA). The initial clinical trials showed beneficial effects on neuropathic pain, cardiac mass and kidney function. However, it has been shown that despite enzyme replacement therapy (ERT), disease complications may still occur [12–14].

Patients who suffer from FD have a lower quality of life (QoL) compared to healthy individuals. Neuropathic pain and anhidrosis are predictors of decreased QoL, presumably as a marker of more severe disease [3]. It has been postulated that ERT has a positive effect on QoL [15, 16]. However, these studies used different measures of QoL and were only reported for small cohorts of patients. Interest in QoL measurements has increased over the past decades, because it is well recognized that, in addition to physical disabilities, emotional and psychological factors play an important role in the lives of patients with FD. Additionally, patient involvement with decision making and assessment of quality of care is increasing. Lastly, QoL measurements are needed for cost-effectiveness analyses, nowadays a requirement for reimbursement of therapy for some governments in the EU [17]. It is therefore important to gain a good understanding of the information available to us now.

This systematic review provides an overview of the current literature with the aim to improve our understanding of the QoL amongst patients with FD and to enhance the appropriate use of QoL instruments in clinical practice. We specifically focus on which QoL measures have been used to determine if these different measures reveal similar results. Furthermore we review the literature on the potential effect of ERT on QoL.

## Methods

### Search strategy and study selection

The following electronic databases have been searched via OvidSP: Medline (1946 till December 10, 2014), Embase (1947 till December 10, 2014) and PsycInfo (1806 till December week 1, 2014). The Cochrane Central Register of Controlled Trials (CENTRAL, accessed December 10, 2014) has been searched as well.

The search terms used were: Fabry disease, quality of life, questionnaires, SF-36, EQ5D, pain measurement, BPI, peds QL, and their synonyms, Mesh terms (Medline) and headings (Embase). No limits were used. Detailed search strategies can be found in Additional file 1.

The title and abstract of all articles obtained by the search were screened to identify studies where quality of life in patients with FD was studied. Reference lists of identified papers were hand searched for additional relevant citations. Original articles published in English, French and German were included. Case reports, case series on less than 5 patients, and review articles were excluded.

### Data extraction

Data were recorded on the type of study (clinical trial, cohort study, before-after study, case series or registry study), number of subjects, gender and age groups (children and/or adults), together with the type of questionnaire used to assess QoL, disease severity and therapy status at the time of QoL assessment.

### Statistical analyses

A meta-analysis was performed on studies reporting SF-36 or RAND-36 results using a fixed effect inverse variance weighting. Meta-analysis of other QoL measurements was not feasible because data were either not given in sufficient detail or QoL instruments were only used in single studies. Articles were included in the meta-analysis when mean domain scores with standard deviations or confidence intervals were provided. Pooled analysis for all studies combined, as well as for subgroups of studies, were performed. Subgroups were defined as: (1) studies performed in the period before ERT was available (untreated, mostly classically affected patients), (2) studies on the effect of ERT that report baseline measurements (untreated patients but with a treatment indication) and (3) studies in which only ERT treated patients were included. Results from the Bodily Pain and General Health subdomains from the RAND-36 were excluded because different scoring algorithm are used for these subdomains.

## Results

**Fig. 1:**
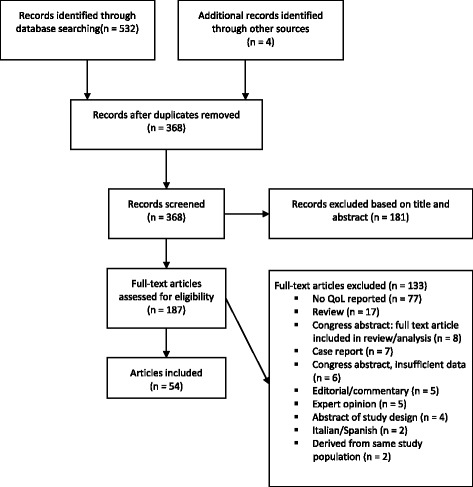
Flow chart of identification, screening and inclusion of articles in the systematic review

The electronic search resulted in 532 publications. Cross-checking reference lists revealed four additional relevant papers. After removal of duplications 368 articles remained. One hundred eighty seven articles were selected based on title and abstract. A total of 54 articles were eligible for inclusion in this review (see Additional file 2) of which 26 reported detailed QoL data. A flow diagram is presented in Fig. 1.

### Questionnaires used to assess QoL in FD

Fifteen different questionnaires have been used to assess QoL in FD populations, amongst which the Short Form (36) Health Survey (SF-36), the EuroQoL five dimensions questionnaire (EQ-5D) and the interference score of the Brief Pain Inventory (BPI) were the most frequently used measures. A short description of these questionnaires is given below.

Other questionnaires used were: the Anderson-Fabry Disease specific questionnaire [7, 18, 19], Child Health Questionnaire [20], Fabry-Specific Pediatric Health and Pain Questionnaire (FPHQP) [21], KINDL [21], PedsQL [22], RAND-36 [23], Rankin scale [24], WHOQOL-100 [25] and four locally developed questionnaires [26–29].

#### SF-36 and RAND-36

The SF-36 [30] questionnaire assesses 8 domains of QoL: (1) Physical Functioning, (2) Role Physical, (3) Bodily Pain, and (4) General Health, (5) Vitality/Energy, (6) Social Functioning, (7) Role Emotional, and (8) Mental Health. SF-36 domain scores range from 0 to 100. The 8 domains can be grouped into two summary scores: the Physical Component Summary (PCS) and the Mental Component Summary (MCS). These scores are computed by multiplying each of the 8 individual SF-36 scores by their specific factor score coefficients. MCS and PCS are norm based summary scores, which are standardised with a T-score transformation resulting in a mean of 50 with a standard deviation of 10. Studies of cross-sectional differences between clinically defined patient groups have suggested a 3 to 5 point change on any SF-36 scale as minimally clinically important difference (MCID) [31]. The RAND-36 [32] is virtually identical to the SF-36 however for the domains General Health and Bodily pain the scoring algorithms are different.

#### EQ-5D and EQ-VAS

The EQ-5D questionnaire is comprised of 5 domains: (1) mobility, (2) self-care, (3) anxiety/depression, (4) usual activities and (5) pain/discomfort [33]. Each domain has 3 levels of severity: (1) no problems, (2) some or moderate problems, and (3) extreme problems. Results from the EQ-5D descriptive system can be converted into a utility score for the calculation of quality-adjusted life years (QALYs) via an algorithm that uses population-based preferences. Utility scores range from −0.11 (all five ED-5D health domains reported extreme problems) to 1 or perfect health (no problems at all five EQ-5D domains), in which zero means dead and negative utility scores represent health states worse than dead. A difference or improvement of 0.074 is considered to be of clinical importance [34]. The EuroQol Visual Analog Scale (EQ-VAS) is a visual analogue scale ranging from 0 to 100 which assesses health state. The minimal important difference is considered to be 7 [35].

### BPI

The Brief Pain Inventory (BPI) [36] has been designed to assess the severity of pain and the impact of pain on daily functions. The latter is reflected by the BPI interference score, which is the average of the following interference subscales: general activity, mood, walking ability, normal work, relations with other people, sleep and enjoyment of life. These subscales are scored from 0 to 10, with an estimated minimal important difference of 1 or 0.5 SD [37].

### QoL in patients with FD versus the general population

**Fig. 2:**
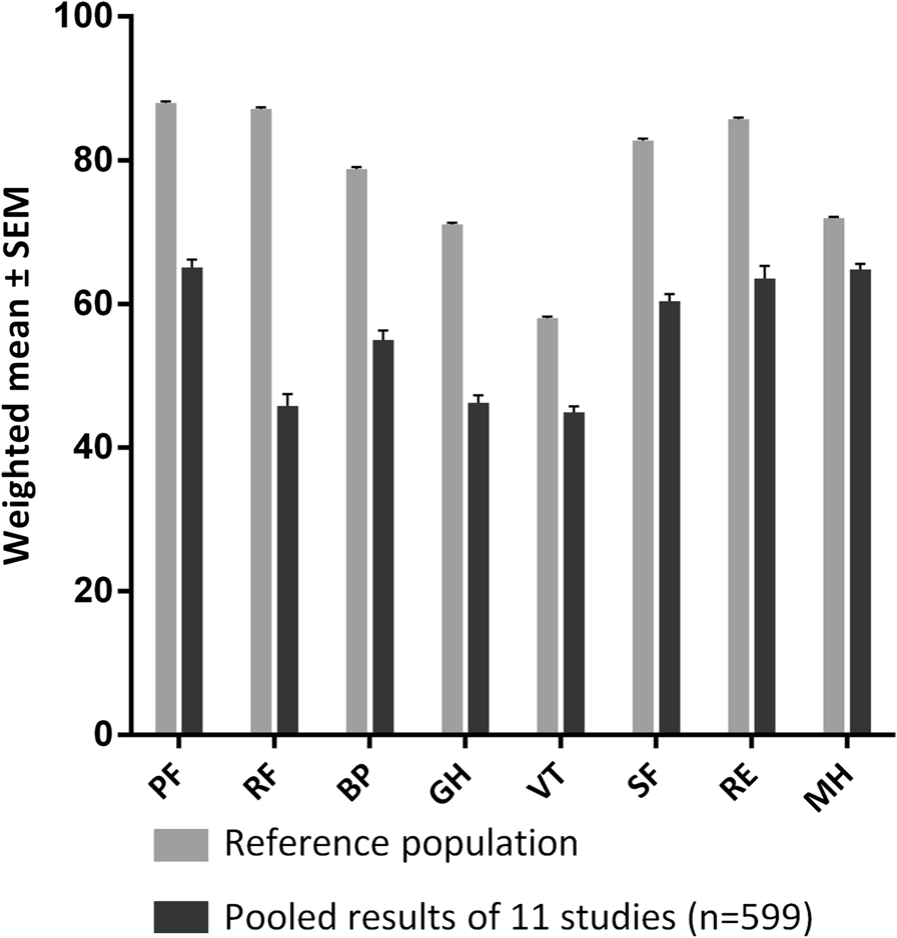
Pooled results of SF-36 subdomain scores. Weighted mean and SEM. Results from treated and untreated, male and female patients. Reference population derived from Jenkinson et al. [45]. *PF* Physical Functioning, *RP* Role Physical, *BP* Bodily Pain, *GH* General Health, *VT* Vitality, *SF* Social Functioning, *RE* Role Emotional, *MH* Mental Health

**Fig. 3:**
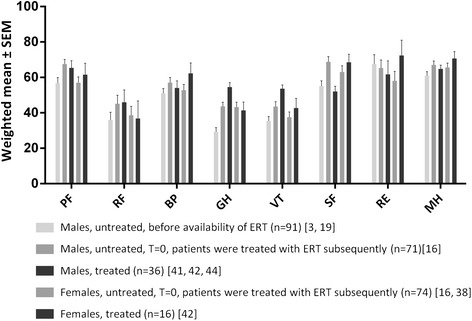
Pooled results of SF-36 subdomain scores stratified by gender and treatment status. Weighted mean and SEM. *PF* physical functioning, *RP* role physical, *BP* bodily pain, *GH* general health, *VT* vitality, *SF* social functioning, *RE* role emotional, *MH* mental health

Eleven studies that investigated QoL in a cohort of FD patients with the SF-36 or the RAND-36 supplied sufficient data for the meta-analysis [3, 16, 19, 23, 38–44]. The results of this meta-analysis (males and females, and treated and untreated patients combined) are depicted in Fig. 2. In general, patients with FD scored worse across all domains compared to the general population [45]. Seven studies reported sufficient data to stratify results by gender and ERT treatment status [3, 16, 19, 38, 41, 42, 44]. Pooled SF-36 scores of these 7 studies are presented in Fig. 3.

Six studies provided the PCS and MCS [19, 22, 38, 39, 43, 46]. Pooled analysis (males and females, and treated and untreated patients combined) revealed a weighted mean of the PCS and MCS of 42.8 (SEM: 0.62) and 48.7 (SEM: 0.52), respectively.

The studies that only mentioned whether or not QoL was better or worse compared to the general population, without providing exact scores, supported these findings; they all showed that QoL in Fabry patients was worse for some or all domains [47–52].

In total, 7 studies used the EQ-5D to compare QoL in patients with FD versus the general population. In 2 studies mean EQ-5D utility scores of 0.66 [53] and 0.56 [19] were reported. The first study was performed in a mixed cohort consisting of males and females, either treated or untreated, while the latter comes from the pre-ERT era and studied only male patients. These scores were both significantly lower than the general population, with 1 of these 2 studies reporting an estimated difference of −0.23 [53]. The third study reported a mean difference of −0.24 in a combined cohort of treated and untreated male and female patients compared with the general population, also a significant difference [54]. Two other studies only mentioned that the EQ-5D score was lower, in a cohort of primarily treated male patients and a cohort of treated female patients without providing any exact data [51, 55]. Finally, two studies reported EQ-VAS scores in mixed cohorts of 21 [40] and 33 [44] untreated and treated FD patients which were significantly lower compared to the general population and matched controls, respectively.

One study used the BPI interference score to measure pain related QoL in male and female patients with FD, either treated or untreated. Compared to age- and gender matched healthy controls, they scored significantly worse (0.4 versus 2.0) [46].

In addition, several other studies suggested a negative influence of FD on QoL, but no comparison with a reference population was made [7, 8, 18, 28, 29, 56, 57].

### The relation between QoL, disease severity and age

Renal disease, disease severity and age are related to QoL in FD. Renal disease impacts on the QoL of FD patients. Significant differences for all SF-36 domain scores except for Mental Health were reported among FD patients with an eGFR of >60 ml/min/1,73 m^2^, patients with an eGFR of <60 ml/min/1,73 m^2^ and patients receiving renal replacement therapy (RRT). Median PCS scores were 49.8 (eGFR >60), 35.9 (eGFR <60) and 29.4 (RRT). The MCS scores were 47.1 (eGFR >60), 49.8 (eGFR <60) and 30.1 (RRT) [58]. Others have shown a negative correlation between MSSI (Mainz Severity Score Index) scores and QoL [39, 55], which was supported by Rombach et al. who defined four disease states; asymptomatic, acroparasthesia/symptomatic, single complication and multiple complications, and found lower EQ-5D utility scores with more severe disease (0.87, 0.76, 0.74, and 0.58 per state, respectively) [59]. Hoffmann et al. investigated specifically gastro-intestinal (GI) complaints amongst male and female patients with FD and unknown treatment status [60]. Using the EQ-5D, patients with GI complaints had a lower EQ-5D score than those without GI complaints.

In line with the relation between disease severity and QoL, higher age has been associated with lower QoL [9, 19, 22, 39]. Qol in males starts to decline at younger age than in female patients as shown by a Fabry Registry (a Genzyme sponsored post-marketing drug registry) study in which males between 18 and 25 years of age had significantly lower SF-36 scores in 6 of 8 subdomains whilst females had normal scores in all but the subscales Bodily Pain and General Health [9]. Above the age of 25, both males and females showed impaired QoL in the subdomains Physical Functioning, Bodily Pain, General Health and Vitality. Females scored better in the Social Functioning domain, while males scored better in the Mental Health subscale [9].

### Effect of ERT on QoL

Two studies reported detailed SF-36 scores to show the effect of ERT on QoL. One Phase IIIB study in 15 female patients with clinical evidence of FD (i.e. involvement of at least 3 organ systems) reported baseline scores [38]. In addition, scores were reported after 13 and 27 weeks of treatment. No control group was included. After 13 weeks of treatment no changes were seen. At week 27, domain scores for Role Physical and General Health were increased, while the other domain scores stayed stable. PCS was 35 (SD: ±12) at baseline, stayed stable after 13 weeks and improved by 6.6 (SD: ± 6.0) after 27 weeks of treatment. MCS was 40 (SD: ±15) at baseline, and similar scores were found after 13 and 27 weeks of treatment.

The second study, based on data from the Fabry Registry, investigated the mean change after 24 months of treatment compared to baseline for males and females separately [16]. Although the Fabry Registry had data of 3128 patients at that time, only for 71 male patients baseline QoL data and three post treatment assessments during a period of 36 months were available. In addition, 59 female patients had baseline and at least 2 post treatment assessments during a period of 24 months. Limited data on genotype, phenotype and disease severity were given. Males showed improvements for all 8 domains after 24 months of treatment with changes from baseline ranging between 4.6 in the Mental Health domain to 14.6 in the Role Physical domain. Females also showed improvements from baseline after 24 months of treatment for Bodily Pain, Vitality, Social Function and Mental Health; 6.1, 7.3, 8.4 and 5.1 respectively. Other domain scores did not change significantly. PCS and MCS scores were calculated at baseline, 12 months and 24 months in the male and female groups, and after 36 months in the male group only. Male PCS and MCS scores were 39 (SD: ±10.9) and 46 (SD: ±10.3) at baseline and did not change significantly after 36 months. Likewise, female PCS scores (baseline score: 37 (SD: ±12.9)) remained stable. MCS scores of females improved from 46 (SD: ±11.8) to 49 (SD: ±11.5) after 24 months.

One study measured mean scores for all domains at baseline, 4 years and 7 years of treatment [61]. Mean SF-36 domain score was 62 (SD: ±19) at baseline and 59 (SD: ±21) after 4 years. After 7 years of treatment the mean score was 57 (SD: ±16) coming from 66 (SD: ±18) at baseline. Changes were not statistically significant, except for the subdomain Social Functioning which worsened significantly after 7 years of therapy. This abstract did not provide patient characteristics, study type, nor detailed baseline and follow-up scores.

Seven studies did not report mean SF-36 domain or summary scores but only mentioned if improvements were observed [48, 51, 62–66]. Five studies mentioned improvements in one or more domains after introduction of ERT, while two studies reported no significant change after 24–36 months of treatment [51, 66]. One of these studies included a placebo group [62]. Both the patients in the placebo and the treatment group showed improvements in the domain Role Physical. In addition, treated patients showed improvement in Role Emotional whereas the placebo patients showed an improvement in the domain Bodily Pain. A study that assessed the relation between treatment duration and QoL reported a negative correlation between time on ERT and SF-36 PCS and MCS scores [22].

Three studies, all using Fabry Outcome Survey (FOS, a Shire sponsored post-marketing drug registry) data, used the EQ-5D to measure the effect of ERT on QoL [53, 54, 67]. Baseline scores were between 0.61 and 0.64, and improvements to 0.74 after 1 year of treatment, and a trend towards improvement to 0.69 in males and 0.72 in females after 4 years of treatment was observed [53, 67]. The third study calculated the difference compared to the general population [54]. At the start of treatment the score was −0.24 lower than the general population and after 5 years of treatment −0.17 below the general population. Wyatt et al. studied the relation between time on ERT and EQ-5D score and found no significant correlation, while EQ-VAS reduced with increased treatment duration [22] A randomised controlled trial in 14 male patients on the efficacy of agalsidase alfa showed a significant difference in change from baseline in BPI interference scores after 24 weeks of ERT, favouring the ERT treatment group (−1,1 vs −0.6) [15]. A second randomized controlled trial did not find an effect of different dosing regimens on this score in the short term [68].

Two studies reported on the effect of home based infusion therapy in comparison to hospital based infusion therapy. A before-after study showed improvement of all SF-36 subscales except Physical Functioning [69]. A cross-sectional study reported less stress and less negative impact on family life after introduction of home treatment [27].

### Effect of the shortage on QoL

In 2009 a temporary worldwide interruption of enzyme supply led to dose reductions or cessation of treatment in groups of FD patients [70]. Three studies investigated the effect of dose decrease or interruption on QoL. Two articles used the SF-36 questionnaire and one article used EQ-5D. The first study using combined data from patients on lower doses of agalsidase beta and patients who switched to agalsidase alfa, showed lower scores for females in the General Health and Vitality domains during the shortage [42]. The second reported no change in MCS and PCS scores after dose reduction of agalsidase beta [71]. The latter, using EQ-5D, showed no change after the switch from agalsidase beta to agalsidase alfa, although a trend towards improvement was seen [72].

### QoL in children with FD

In a paediatric cohort of 87 children (boys and girls combined) the mean EQ-5D utility score was 1.00 (SD ±0.0), the mean of the interference score of the BPI was 0.76 (SD 1.47), and the KINDL showed a moderate impact on QoL and daily life, with the most severely affected domains being personal feeling, family and friends [21]. In 22 boys and girls of whom seven received ERT the total PedsQL score as well as the subscales physical functioning and school functioning decreased with age, while no relation with time on ERT was found [22]. BPI interference scores decreased after introduction of ERT in 13 children [73]. In nine children (age <10) all subscales of the Child Health Scores were lower, but only bodily pain and mental health were significantly different from a healthy control population. Children above the age of 10 years only scored worse on the bodily pain domain compared to the control population [20]. Thirty-six adolescent patients scored worse on the SF-36 compared to general population; boys reported decreased QoL in all subscales, except for Role Emotional, while females scored worse in the subscales Bodily Pain and General Health [74].

### QoL in FD vs other chronic illnesses

Street et al. investigated data from female FD patients and compared them with cohorts with multiple sclerosis (MS) and rheumatoid arthritis (RA) using the RAND-36 [23]. Females with FD scored better on the Physical Functioning domain than MS and RA patients (67 versus 37 and 51, respectively). Similar scores were found in patients with FD and patients with MS in the domains Role Emotional, Energy and Emotional Well-being patients with FD while RA patients scored better. Social functioning and Role Physical scores in patients with FD were comparable to those in patients with RA, while patients with MS scored worse in these domains. Pain scores of FD patients were worse than those in MS patients but better than those in RA patients (62 versus 74 and 56, respectively). General health is lower in patients with FD than in patients with MS and RA (45 versus 53 and 51, respectively). A second study compared patients with FD to patients with RA, MS, central neuropathic pain and Gaucher disease (GD). Baseline SF-36 scores were similar to MS and GD patients. General Health and Vitality scores in patients with FD were comparable to those in RA and central neuropathic pain patients [16]. A third study reported substantially lower General Health, Vitality, Social Functioning, Role Emotional and Mental Health domain scores in female patients with FD compared to patients with RA [38].

In addition, patients with FD have been compared to severe hemophiliacs [19]. MCS and EQ-VAS scores were lower in patients with FD; PCS scores and EQ-5D were similar in both populations. Finally, a study using WHOQoL-100 compared QoL of FD patients with that of PKU patients [25]. General QoL as well as the physical, independence, facet medication domains were lower in patients with FD. The scores in the psychological, spiritual and environmental domains were similar.

## Discussion

This systematic review of quality of life in Fabry patients from 54 articles and abstracts has led to two major findings. Firstly, a consistent finding from all studies is that Fabry patients suffer from a considerably worse quality of life as compared with the general population. This was found for all domains in the SF-36 and in the EQ-5D questionnaires. Secondly, the studies on the effect of ERT on QoL are inconclusive.

Both the SF-36 and EQ-5D revealed that patients with FD clearly have lower QoL scores in comparison with the healthy population. However, in the interpretation of these results, some of the study characteristics need to be taken into consideration; firstly, disease severity is rarely comprehensively reported and if reported, the data varies between studies. Rombach et al. showed that disease severity plays an important role for measuring QoL and should therefore be taken into account when measuring QoL scores [59]. This is further supported by the finding of a correlation between the MSSI (a measure for disease severity) and QoL [39, 55]. In addition, more severe kidney disease has been shown to lead to reduced QoL, in particular after initiation of renal replacement therapy. [58]. Also, no studies on the difference in QoL between patients with either classical or non-classical FD have been performed, although this would have been interesting considering their different disease courses. The influence of phenotype is illustrated by Gold et al., who measured SF-36 scores for untreated male patients before the introduction of ERT. In these severely, mostly classically affected males, domain scores ranged between 24 and 61, which is worse compared to QoL scores found after the introduction of ERT, even if only baseline scores (prior to start of ERT) are considered. This difference can be partly explained by the inclusion of non-classical patients in the more recent studies. In addition, most studies investigated a Fabry population consisting of both males and females. As noted by Wilcox et al. differences are observed between males and females and at what age the quality of life starts to decline for either gender [9]. Both are factors that need to be taken into consideration. Thirdly, many of the cohorts studied consisted of treated and untreated patients together. More studies in subgroups of patients are needed to gain a better insight into the influence of phenotype, gender and treatment on QoL.

If specifically looking at the effect of ERT on QoL, only a limited number of studies reported baseline and follow-up data in detail, showing different results. One study of only women, with a small sample size and without a control arm discovered a very minor change after 27 weeks. Another study reported a minimal improvement in BPI interference score after 24 weeks, although it should be noted that baseline BPI scores were different between both treatment arms and decreased in both groups [15]. One might argue that 6 months is too short to detect any effect from ERT on QoL scores. However, a third study showed no change in subdomains of the SF-36 after 4 and 7 years of therapy except for Social Functioning, which worsened after 7 years of therapy [61]. Another explanation could be that the questionnaires are not sensitive enough to show a clear effect in patients with FD. Baumstarck et al. demonstrated that generic questionnaires often are more suitable for universal applications where QoL is compared in different populations, while disease specific instruments focus on particular health problems and are more sensitive for detecting and quantifying small changes [75, 76]. This would suggest that a Fabry specific QoL questionnaire would provide a more sensitive tool to investigate the effects of ERT on the QoL. At this point no validated FD specific QoL questionnaire exists and it would be worthwhile to develop such a questionnaire for this patient group. The studies based on data from the Fabry Registry or Fabry Outcome Survey all showed an improvement [16, 53, 54, 67]. Despite being large, these registries have their shortcomings as has been published by Hollak et al. [77]. Follow-up data on QoL of only a very small percentage of patients enrolled in these registries were available for the analysis making the results susceptible to selection bias. Furthermore, the limited information on genotype, phenotype and disease severity of the patients in these registry studies makes comparison between cohorts impossible. Finally, differences before and after therapy are small, especially when comparing to the 3–5 point (SF-36) or 0.074 (EQ-5D) minimally clinically important difference (MCID). However, whether the standard MCID’s are applicable to FD can be questioned. For example, Wyrwich et al. demonstrated that three different expert panels provided three different Clinically Important Differences for three different diseases; chronic obstructive pulmonary disease (COPD), asthma, and heart disease, respectively [78]. This would imply that a FD specific MCID needs to be defined for an optimal interpretation of the results. Altogether, no clear answer can be given whether ERT has a positive or negative effect on standard QoL scores in patients with FD.

Apart from the need for more sensitive questionnaires and disease specific MCID’s, another important caveat is the lack of QoL data from untreated patients with similar disease severity as those treated with ERT. Only two short-term placebo-controlled trials of ERT in FD with QoL as a secondary outcome measure have been performed. One of those studies revealed significant improvement in both placebo and treated arms [62], while the other showed a small improvement in BPI interference score compared to the placebo group [15]. The 3 studies published on the effects of the shortage did provide us with an opportunity to see how ERT affected the QoL once the preparation or dose was changed. However, these studies were of relatively short duration and no clear conclusion can be drawn from the results. Two studies reported stable QoL scores, while one study established a decline in two subdomains of the SF-36, only in female patients [42]. Whether anxiety of patients due to the situation of shortage played a role is unknown as well.

Finally, several national governments currently ask for cost effectiveness analyses to aid in reimbursement decisions. SF-36 and EQ-5D utility scores play a central role in these analyses. Based on these scores, QALY’s are calculated and subsequently used to obtain costs per QALY. This development stresses the importance for the collection of high quality QoL data both before and during treatment.

## Conclusion

Patients who suffer from FD have a considerably lower QoL compared to the general population; this was shown in two generic questionnaires, the EQ-5D and the SF-36. The effect of ERT is however inconclusive; small cohorts, lack of data and limited natural history data hamper a definite conclusion. We propose that a FD specific QoL questionnaire is developed to accurately monitor patients who suffer from this disease.

## Additional files

Additional file 1:
**Search strategy. Detailed description of the search strategy used.**
Additional file 2:
**Overview of included studies. Table of included studies.**

## Abbreviations

BPI: Brief pain inventory
ERT: Enzyme replacement therapy
GB3: Globotriaosylceramide
EQ-5D: 5 Dimension euroqol questionnaire
EQ-VAS: EuroQol visual analog scale
FD: Fabry disease
FOS: Fabry Outcome Survey
MSSI: Mainz severity score index
QoL: Quality of life
SF-36: Short form (36) health survey
