# Supplementary material for: Quality of life in patients with Fabry disease: a systematic review of the literature
Source: Orphanet J Rare Dis. 2015 Jun 16;10:77. doi: 10.1186/s13023-015-0296-8 (PMC4501376; doi:10.1186/s13023-015-0296-8)
Supplement: Additional file 1: — Search strategy. Detailed description of the search strategy used. [file 13023_2015_296_MOESM1_ESM.docx]

## additional file 1 Search strategy

| **EMBASE (1947 – December 10, 2014), via OvidSP** | | |
| --- | --- | --- |
| ***Fabry disease*** | | |
| 1 | exp Fabry Disease/ | |
| 2 | fabry*.ti,ot,ab,hw,kw. | |
| 3 | (angiokeratoma adj3 diffusum).ti,ot,ab,kw,hw. | |
| 4 | (diffuse angiokeratoma*).ti,ot,ab,kw,hw. | |
| 5 | (galactosidase adj3 deficiency).ti,ot,ab,kw,hw. | |
| 6 | exp alpha-Galactosidase/ | |
| 7 | (alpha galactosidase).ti,ot,ab,kw,hw. | |
| 8 | (GLA adj3 deficiency).ti,ot,ab,kw,hw. | |
| ***Enzyme replacement therapy*** | | |
| 9 | replagal.ti,ot,ab,kw,hw,tn. | |
| 10 | exp agalsidase alfa/ | |
| 11 | fabrazyme.ti,ot,ab,kw,hw,tn. | |
| 12 | exp agalsidase beta/ | |
| 13 | agalsidase.ti,ot,ab,kw,hw,tn. | |
| ***Quality of life*** | | |
| 14 | exp "Quality of Life"/ | |
| 15 | (quality adj3 life).ti,ot,ab,kw,hw. | |
| 16 | (QoL or hrQoL).ti,ot,ab,kw,hw. | |
| ***Questionnaires*** | | |
| 17 | exp Questionnaires/ | |
| 18 | questionnair*.ti,ot,ab,kw,hw. | |
| ***Quality of life questionnaires*** | | |
| 19 | exp Short Form 36/ | |
| 20 | (SF-36 or SF36 or sf 36 or short form 36 or short form-36).ti,ot,ab,kw,hw. | |
| 21 | (RAND 36 or RAND36 or RAND-36 or (RESEARCH adj2 DEVELOPMENT 36) or RAND-36 item Health Survey or RAND 36 item Health Survey).ti,ot,ab,kw,hw. | |
| 22 | (EQ5D or EQ-5D or EuroQol or Euro-QoL).ti,ot,ab,kw,hw. | |
| 23 | (QOLS or Quality of life scale).ti,ot,ab,kw,hw. | |
| 24 | (PedsQL OR (pediatric quality of life)).ti,ot,ab,kw,hw. | |
| 25 | (FPHPQ OR (Fabry-specific Pediatric Health and Pain Questionnaire)).ti,ot,ab,kw,hw. | |
| 26 | ((AFD specific questionnaire) or (Fabry* and specific questionnaire) or (AFD specific questions) or (Fabry* and specific questions)).ti,ot,ab,kw,hw. | |
| 27 | ((Disease severity scoring system) OR (DS3)).ti,ot,ab,kw,hw. | |
| ***Pain questionnaires*** | | |
| 28 | exp pain assessment/ | |
| 29 | (BPI or (brief pain inventory)).ti,ot,ab,kw,hw. | |
| 30 | exp Brief Pain Inventory/ | |
| 31 | exp McGill Pain Questionnaire/ | |
| 32 | (McGill pain).ti,ot,ab,kw,hw. | |
| 33 | (FPQ or (Fabry pain questionnaire)).ti,ot,ab,kw,hw. | |
| ***Combined*** | | |
| 34 | or/1-13 | *Combined concepts Fabry and enzyme replacement therapy* |
| 35 | or/14-33 | *Combined concepts Quality of life and questionnaires* |
| 36 | 34 AND 35 | *Combined search, results used in the current study* |
| **MEDLINE (1946 – December 10, 2014) , via OvidSP** | | |
| ***Fabry disease*** | | |
| 1 | exp Fabry Disease/ | |
| 2 | fabry*.ti,ot,ab,kf. | |
| 3 | (angiokeratoma adj3 diffusum).ti,ot,ab,kf. | |
| 4 | (diffuse angiokeratoma*).ti,ot,ab,kf. | |
| 5 | (galactosidase adj3 deficiency).ti,ot,ab,kf. | |
| 6 | exp alpha-Galactosidase/ | |
| 7 | (alpha galactosidase).ti,ot,ab,kf. | |
| 8 | (GLA adj3 deficiency).ti,ot,ab,kf. | |
| ***Enzyme replacement therapy*** | | |
| 9 | replagal.ti,ot,ab,kf,nm. | |
| 10 | fabrazyme.ti,ot,ab,kf,nm. | |
| 11 | agalsidase.ti,ot,ab,kf,nm. | |
| ***Quality of life*** | | |
| 12 | exp "Quality of Life"/ | |
| 13 | (Quality adj3 life).ti,ot,ab,kf. | |
| 14 | (QoL or hrQoL).ti,ot,ab,kf. | |
| ***Questionnaires*** | | |
| 15 | exp Questionnaires/ | |
| 16 | Questionnair$.ti,ot,ab,kf. | |
| ***Quality of life questionnaires*** | | |
| 17 | (SF-36 or SF36 or sf 36 or short form 36 or short form-36).ti,ot,ab,kf. | |
| 18 | (RAND 36 or RAND36 or RAND-36 or (RESEARCH adj2 DEVELOPMENT 36) or RAND-36 item Health Survey or RAND 36 item Health Survey).ti,ot,ab,kf. | |
| 19 | (EQ5D or EQ-5D or EuroQol or Euro-QoL).ti,ot,ab,kf. | |
| 20 | (QOLS or Quality of life scale).ti,ot,ab,kf. | |
| 21 | (PedsQL OR (pediatric quality of life)).ti,ot,ab,kf. | |
| 22 | (FPHPQ OR (Fabry-specific Pediatric Health and Pain Questionnaire)).ti,ot,ab,kf. | |
| 23 | ((AFD specific questionnaire) or (Fabry* and specific questionnaire) or (AFD specific questions) or (Fabry* and specific questions)).ti,ot,ab,kf. | |
| 24 | ((Disease severity scoring system) OR (DS3)).ti,ot,ab,kf. | |
| ***Pain questionnaires*** | | |
| 25 | exp Pain Measurement/ | |
| 26 | (BPI or (brief pain inventory)).ti,ot,ab,kf. | |
| 27 | (McGill pain).ti,ot,ab,kf. | |
| 28 | (FPQ or (Fabry pain questionnoire)).ti,ot,ab,kf. | |
| ***Combined*** | | |
| 29 | or/1-11 | *Combined concepts Fabry and enzyme replacement therapy* |
| 30 | or/12-28 | *Combined concepts Quality of life and questionnaires* |
| 31 | 29 AND 30 | *Combined search, results used in the current study* |
| **PsychInfo (1806 till December week 1, 2014) , via OvidSP** | | |
| ***Fabry disease*** | | |
| 1 | fabry*.ti,ab. | |
| 2 | (angiokeratoma adj3 diffusum).ti,ab. | |
| 3 | (diffuse angiokeratoma*).ti,ab. | |
| 4 | (galactosidase adj3 deficiency).ti,ab. | |
| 5 | (alpha galactosidase).ti,ab. | |
| 6 | (GLA adj3 deficiency).ti,ab. | |
| ***Enzyme replacement therapy*** | | |
| 7 | replagal.ti,ab. | |
| 8 | fabrazyme.ti,ab. | |
| 9 | agalsidase.ti,ab. | |
| ***Quality of life*** | | |
| 10 | exp "Quality of Life"/ | |
| 11 | (Quality adj3 life).ti,ab. | |
| 12 | ((QoL) or (hrQoL)).ti,ab. | |
| ***Questionnaires*** | | |
| 13 | exp Questionnaires/ | |
| 14 | Questionnair*.ti,ab. | |
| ***Quality of life questionnaires*** | | |
| 15 | (SF-36 or SF36 or sf 36 or short form 36 or short form-36).ti,ab,tm. | |
| 16 | (RAND 36 or RAND36 or RAND-36 or (RESEARCH adj2 DEVELOPMENT 36) or RAND-36 item Health Survey or RAND 36 item Health Survey).ti,ab,tm. | |
| 17 | (EQ5D or EQ-5D or EuroQol or Euro-QoL).ti,ab,tm. | |
| 18 | (QOLS or Quality of life scale).ti,ab,tm. | |
| 19 | (PedsQL OR (pediatric quality of life)).ti,ab,tm. | |
| 20 | (FPHPQ OR (Fabry-specific Pediatric Health and Pain Questionnaire)).ti,ab,tm. | |
| 21 | ((AFD specific questionnaire) or (Fabry* and specific questionnaire) or (AFD specific questions) or (Fabry* and specific questions)).ti,ab,tm. | |
| 22 | ((Disease severity scoring system) OR (DS3)).ti,ab,tm. | |
| ***Pain questionnaires*** | | |
| 23 | (BPI or (brief pain inventory)).ti,ab,tm. | |
| 24 | (McGill pain).ti,ab,tm. | |
| 25 | (FPQ or (Fabry pain questionnaire)).ti,ab,tm. | |
| ***Combined*** | | |
| 26 | or/1-9 | *Combined concepts Fabry and enzyme replacement therapy* |
| 27 | or/10-25 | *Combined concepts Quality of life and questionnaires* |
| 28 | 26 AND 27 | *Combined search, results used in the current study* |
| **CENTRAL (accessed December 10, 2014)** | | |
| ***Fabry disease*** | | |
| 1 | fabry*:ti,ab,kw | |
| 2 | diffuse angiokeratoma:ti,ab,kw | |
| 3 | galactosidase deficiency:ti,ab,kw | |
| 4 | GLA deficiency:ti,ab,kw | |
| 5 | MeSH descriptor: [Fabry Disease] explode all trees | |
| 6 | MeSH descriptor: [alpha-Galactosidase] explode all trees | |
| ***Enzyme replacement therapy*** | | |
| 7 | replagal:ti,ab,kw | |
| 8 | fabrazyme:ti,ab,kw | |
| 9 | agalsidase:ti,ab,kw | |
| ***Quality of life*** | | |
| 10 | MeSH descriptor: [Quality of Life] explode all trees | |
| 11 | quality near life:ti,ab,kw | |
| 12 | (QoL) or (hrqol):ti,ab,kw | |
| ***Questionnaires*** | | |
| 13 | MeSH descriptor: [Questionnaires] explode all trees | |
| 14 | questionnaire*:ti,ab,kw | |
| ***Quality of life questionnaires*** | | |
| 15 | ((SF36) or (SF-36) or (short form 36) or (short form-36)):ti,ab,kw | |
| 16 | ((RAND 36) or (RAND36) or (RAND-36) or (RESEARCH near DEVELOPMENT 36) or (RAND-36 item Health Survey) or (RAND 36 item Health Survey)):ti,ab,kw | |
| 17 | (EQ5D or EQ-5D or EuroQol or Euro-QoL):ti,ab,kw | |
| 18 | (QOLS or Quality of life scale):ti,ab,kw | |
| 19 | (PedsQL or "pediatric quality of life"):ti,ab,kw | |
| 20 | (FPHPQ or "Fabry-specific Pediatric Health and Pain Questionnaire"):ti,ab,kw | |
| 21 | ("AFD specific questionnaire" or (Fabry* and specific questionnaire) or (AFD specific questions) or (Fabry* and specific questions)):ti,ab,kw | |
| 22 | ("Disease severity scoring system" or DS3):ti,ab,kw | |
| ***Pain questionnaires*** | | |
| 23 | MeSH descriptor: [Pain Measurement] explode all trees | |
| 24 | BPI:ti,ab,kw | |
| 25 | Brief pain questionnaire:ti,ab,kw | |
| 26 | McGill pain:ti,ab,kw | |
| 27 | FPQ or "Fabry pain questionnaire":ti,ab,kw | |
| ***Combined*** | | |
| 28 | or/1-9 | *Combined concepts Fabry and enzyme replacement therapy* |
| 29 | or/9-27 | *Combined concepts Quality of life and questionnaires* |
| 30 | 29 AND 30 | *Combined search, results used in the current study* |
