# Supplementary material for: Quality of life in patients with Fabry disease: a systematic review of the literature
Source: Orphanet J Rare Dis. 2015 Jun 16;10:77. doi: 10.1186/s13023-015-0296-8 (PMC4501376; doi:10.1186/s13023-015-0296-8)
Supplement: Additional file 2: — Overview of included studies. Table of included studies. [file 13023_2015_296_MOESM2_ESM.docx]

## **additional file 2** Overview of included studies

| **Study** | **design** | **Controls** | **Questionnaire** | **No of patients (males) in whom QoL is measured** | **ERT** | **Disease severity** |
| --- | --- | --- | --- | --- | --- | --- |
| Baehner et al. (2003)† | before after study, non-comparative | General population,  RA patients | SF-36 | 15 (0) | 100% | 67% >6 affected organ systems. Creatinine clearance varied from 65 to 73 ml/min/1.73m^2^. Average LVMi at baseline: 148 g/m^2^ |
| Barba-Romero et al. (2011)‡ | case series | N/A | EQ-5D | 10 (2)  [=12% of patients included in study] | ♀ 94%  ♂ 48% | Not available for QoL subgroup.  Overall: mean MSSI: ♀7, ♂14.5 |
| Beck et al. (2013) † | before after study, non-comparative | N/A | SF-36 | 69 (?) | 100% | Not available |
| Bouwman et al. (2011) † | cross sectional, comparative | General population | SF-36 | 28 (9) | 64% | Mean MSSI: ♀4 ♂16. Proteinuria ♀5% ♂11%. WML ♀16% ♂33%. LVH ♀16% ♂33%. |
| Bouwman et al. (2012) | case control | Age matched controls | locally developed questionnaire | 62 (0) | Unknown | Not available |
| Buechner et al. (2008) | case series | N/A | EQ-5D, Rankin scale | 43 (25) | 56% | Renal dysfunction ♀28% ♂ 76%. Cardiac involvement ♀ 39% ♂ 68%. Stroke ♀28% ♂24% |
| Cazzorla et al. (2012) | cross sectional, comparative | Other inherited metabolic diseases | WHOQOL-100 | 13 (?) | Yes,  % unknown | Not available |
| Deegan et al. (2006) | case series | N/A | EQ-5D | 130 (0)  [=43% of patients included in study] | Yes,  % unknown | Not available for QoL subgroup.  Overall: Stroke 7%, LVH 26%, proteinuria 35% |
| Duning et al. (2012) † | cross sectional, comparative | General population | SF-36 | 49 (27) | Unknown | MSSI range 0-51, mean 15. CVA 10%. Cardiac abnormalities 27%, proteinuria 29% |
| Eng et al. (2001) | NRCT | FD (different dosing regimens) | SF-36 | 15 (15) | 100% | Classically affected, nu further information. |
| Eng et al. (2001) | RCT | FD, placebo | SF-36 | 58 (56) | 50% | Mean GFR: ERT 83, placebo 96 |
| Eto et al. (2005) | before after study, non-comparative | N/A | SF-36, BPI interference | 13(13) | 100% | Mean creatinine 93ɥmol/L |
| Faggiano et al. (2006) | case control | Age and gender matched controls | SF-36 | 18 (9) | 55% | “classic” male patients. Overall 28% CKD stage III-V, LVH 72% |

| Geevasing et al. (2012) † | cross sectional, non-comparative | N/A | BPI interference | 13 (10) | 46% | Mean MSSI 16 |
| --- | --- | --- | --- | --- | --- | --- |
| Germain et al. (2007) | before after study, non-comparative | N/A | SF-36 | 58 (56) | 100% | All patients had normal GFR |
| Ghali et al. (2012) ‡ | Cohort study, retrospective | FD patients (different treatment groups) | SF-36 | 40 (32) | 100% | Not available for QoL subgroup  Overall: GFR 93, LVH 7%, small cerebral infarction 7% |
| Gibas et al. (2006) | cross sectional study | N/A | locally developed questionnaire | 96 (45) | Unknown | Not available |
| Gold et al. (2002) † | cross sectional study, comparative | General population | SF-36 | 53 (53) | - | Not available |
| Gupta et al (2005) | Cross sectional study | N/A | BPI | 50 (50)  [=88% of patients included in study] | - | MeanGFR : 93, |
| Hoffmann et al. (2005) †‡ | before-after study | N/A | EQ-5D, BPI | 120 (73) | 100% | Not available |
| Hoffmann et al. (2007) ‡ | case series | N/A | EQ-5D | 262 (133)  [=35% of patients included in study] | Unknown | Not available |
| Hoffmann et al. (2007) ‡ | case series | N/A | BPI | 108 (41)  [=32% of patients included in study] | Unknown | Not available |
| Hopkin et al. (2008) ‡ | case series | N/A | SF-36 | 352 (194)  [36 (10)] | 40% | Not available |
| Hughes et al. (2011) †‡ | before after study | N/A | EQ-5D | [60 (37)  [=24% of patients included in study] | 100% | Not available for QoL subgroup.  Mean LVMi ♀ 48.2 ♂ 54.7 g/m^2.7^, mean eGFR ♀ 71.8 ♂ 88.2 |
| Hughes et al. (2013) † | RCT (cross over) | Cross over design | EQ-5D | 19 (13) | 100% | Not available |
| Kantola et al. (2012) | case series  (congress abstract) | N/A | SF-36 | 16 (?) | 100% | Not available |
| Koskenvuo et al. (2008) | open label prospective follow up study | N/A | SF-36 | 9 (5) | 100% | Mildly impaired to normal renal function, LVH 62%, atrial fibrillation present in 11% |
| Low et al. (2007) † | cross sectional study, comparative / before after study | General population | SF-36 | 21 (19) | 76% | 38% ESRD, 10% stroke |

| MacDermot et al. (2001) | cross sectional study | N/A | AFD specific questionnaire | 46 (46)  [=47% of patients included in study] | - | Not available for QoL subgroup.  Overall: ESRD 31%, LVH 88%, TIA/CVA 24% |
| --- | --- | --- | --- | --- | --- | --- |
| MacDermot et al. (2001) | cross sectional study | N/A | AFD specific questionnaire | 29 (0)  [=48% of patients included in study] | - | Not available for QoL subgroup.  Overall: ESRD 3%, 47%, LVH 19%, TIA/CVA 22% |
| Metha et al. (2009) †‡ | case series | General population | EQ-5D | [51 (?)  [=28% of patients included in study] | 100% | Not available for QoL subgroup.  Overall: mean eGFR 85 , mean LVMi 58 g/m^2.7^ |
| Milligan et al. (2006) | case series | N/A | locally developed questionnaire | 20 | 100% | Not available |
| Miners et al. (2002) † | cross sectional study, comparative | General population | SF-36, EQ-5D, AFD specific questionnaire | 38 (38) | - | Heart symptoms 74%, stroke 13%, ESRD 16%. Classically affected males. |
| Morier et al. (2010) | cross sectional study | N/A | locally developed questionnaire | 23 (8) | - | Not available |
| Oliveira et al. (2013) † | cross sectional, comparative | Elderly population | SF-36 | 14 (10) | 71% | Not available |
| Quinn et al. (2010) | cross sectional study (congress abstract) | N/A | SF-36 | 32 (18) | Unknown | Not available |
| Ramaswami et al. (2012) †‡ | validation study | N/A | EQ-5D, BPI interference, FPHPQ, KINDL | 87 (44) | 57% | 93% MSSI <20 |
| Ramaswami et al. (2007) | before after study | N/A | BPI interference | 13 (9) | 100% | Acroparesthesia 77%, 15% asymptomatic |
| Ries et al. (2005) | case control | General population | Child health questionnaire | 25 (25) | Unknown | Renal function, urinary protein excretion, and cardiac function and structure were normal for the majority of patients. |
| Rombach et al. (2013) † | HTA / cohort study | N/A | EQ-5D | 100 (48) | 58% | Stratified by disease severity |
| Schermuly et al. (2011) † | case control | Age, gender and educational matched controls | SF-36, BPI interference | 25 (10) | 80% | Mild to moderate disease involvement, mean MSSI 21 |
| Schiffman et al. (2001) † | RCT | Placebo controlled | 0 | 26 (26) | 54% | 19 patients >4 organ systems involved |
| Smid et al. (2011) † | before after study | N/A | SF-36 | 35 (17) | 100% | Not available |
| Street et al. (2006) † | cross sectional, comparative | General population, MS patients, RA patients | RAND-36 | 202 (0) | Unknown | Renal insufficiency 17%, TIA 20%, Stroke 8%, arrhythmia 44%, LVH 18%, proteinuria 38%. |
| Torvin et al. (2009) | cross sectional | General population | SF-36 | 19 (0) | 53% | Not available |
| Tsuboi et al. (2012) † | before after study | N/A | EQ-5D | 11 (4) | 100% | Mean eGFR 90, LVMi (g/m^2.7^) 58, MSSI 22 |
| Vedder et al. (2007) | Case series | N/A | SF-36 | 71 (27) | 45% | CKD III-V ♀ 5% ,♂ 30%, LVH ♀ 63% ,♂ 45% |
| Wagner et al. (2014) † | retrospective cohort study | FD patients with&without renal impairment | SF-36 | 96 (46) | 45% | Stratified by disease severity |
| Wang et al. (2007) † | prospective cohort study | General population | SF-36, BPI interference | 19 (0)  [=45% of patients included in study] | 26% | Not available for QoL subgroup.  Overall: stroke 22%, LVH 24%, ESRD 13%, proteinuria 56% |
| Watt et al. (2010) †‡ | before after study | Patients with other chronic disease | SF-36 | 130 (71) | 100% | Renal events ♀ 0% ,♂ 7%, cardiovascular events ♀ 25% ,♂ 23%, stroke ♀ 1% ,♂ 9% |
| Wilcox et al. (2008) †‡ | case series | General population | SF-36 | 558 (368)  [=25% of patients included in study] | Unknown | Stroke ♀ 4% ,♂ 7%, LVH ♀ 18.2% ,♂ 21.6%, CKD III-V ♀ 34% ,♂ 19% |
| Wilcox et al. (2004) | before after | N/A | SF-36 | 58 (56) | 100% | All patients had normal GFR |
| Wyatt et al. (2012)  Adults *† | prospective cohort study | N/A | SF-36, EQ-5D, BPI | 289 (120) | 73% | Proteinuria ♀ 19%, ♂ 48% , mean LVMi g/m^2^ 107 , mean eGFR in patients without proteinuria: 85 and with proteinuria 83 |
| Wyatt et al. (2012)  Children *† | prospective cohort study | N/A | pedsQL | 22 (11) | 32% | LVMi g/m^2^ 68 g/m^2^ , mean GFR in patients without proteinuria: 110 and with proteinuria 81 |
| Zuraw et al. (2011) † | cross sectional | General population | SF-36, EQ-5D | 32 (20) | 63% | Not available |
| † study reported detailed information on QoL. ‡ FOS or Fabry Registry based study. RCT = randomised controlled trial, NRCT = non-randomised controlled trial, N/A = not applicable, RA = rheumatoid arthritis, MS = multiple sclerosis. MSSI = Mainz severity index, GFR = glomerular filtration rate, in ml/min/1.73m^2^, CKD = chronic kidney disease, ESRD = end stage renal disease, LVH = left ventricular hypertrophy, LVMi = left ventricular mass index, WML = white matter lesions | | | | | | |
